# Unpredictable environments lead to the evolution of parental neglect in birds

**DOI:** 10.1038/ncomms10985

**Published:** 2016-03-29

**Authors:** Shana M. Caro, Ashleigh S. Griffin, Camilla A. Hinde, Stuart A. West

**Affiliations:** 1 Department of Zoology, University of Oxford, South Parks Road, Oxford OX1 3PS, UK; 2 Behavioural Ecology Group, Department of Animal Sciences, Wageningen University, PO Box 338, 6700 AH Wageningen, The Netherlands

## Abstract

A nest of begging chicks invites an intuitive explanation: needy chicks want to be fed and parents want to feed them. Surprisingly, however, in a quarter of species studied, parents ignore begging chicks. Furthermore, parents in some species even neglect smaller chicks that beg more, and preferentially feed the biggest chicks that beg less. This extreme variation across species, which contradicts predictions from theory, represents a major outstanding problem for the study of animal signalling. We analyse parent–offspring communication across 143 bird species, and show that this variation correlates with ecological differences. In predictable and good environments, chicks in worse condition beg more, and parents preferentially feed those chicks. In unpredictable and poor environments, parents pay less attention to begging, and instead rely on size cues or structural signals of quality. Overall, these results show how ecological variation can lead to different signalling systems being evolutionarily stable in different species.

## Abstract (web summary)

The response of parents to offspring begging behaviour is hugely variable in birds, but what mediates this response is not known. In a meta-analysis across 143 species, Caro *et al*. show that variation in offspring begging and parental care is a function of environmental quality and predictability.

## Graphical abstract

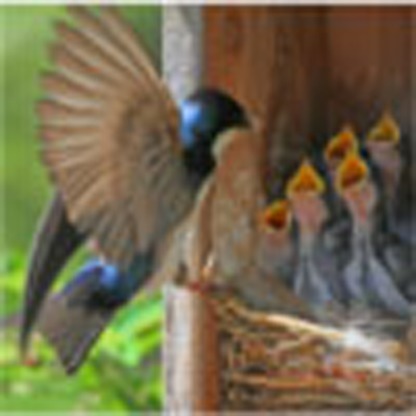

In many species, including our own, the production of offspring represents the most energetically demanding stage of an animal's life. Raising a brood successfully puts a metabolic demand on breeding birds that is the equivalent to a human cycling the Tour de France^1^. Success or failure often depends on parents' ability to determine which offspring to invest in, when to invest in them and how much to invest. Offspring attempt to influence the feeding behaviour of their parents by begging for food through a variety of mechanisms, including vocal calls, behavioural displays and physical structures^2^.

Different species, however, appear to beg and respond to begging in different ways (Fig. 1)^3^,^4^,^5^,^6^,^7^,^8^,^9^. In many species, such as the tree swallow, smaller nestlings beg more, and are preferentially fed by their mothers^10^,^11^. In other species, such as the hoopoe, mothers sometimes force food into the beaks of larger, silent chicks, ignoring the persistent begging from their smaller offspring^12^. In many siblicidal species, such as the blue-footed booby, the largest offspring beg and are fed the most^13^.

Evolutionary theory has been unable to account for this diversity across species, as highlighted by Mock *et al*.^3^. The dominant paradigm, ‘signal of need', predicts that chicks in worse condition beg with greater intensity and that parents respond to this begging^3^,^14^,^15^,^17^. However, these signal of need models assume that parents are trying to rear all their offspring and that offspring in better condition reduce their begging^3^,^4^,^9^,^14^,^17^. This is clearly not the case in species where parents let the neediest offspring starve and offspring in better condition beg more^3^,^13^,^18^,^19^,^20^,^21^. The pattern in those species may be better explained by ‘signal of quality' models^3^,^20^,^21^. However, neither signal of need nor quality models predict that parents should flexibly ignore begging, as the hoopoe does^12^,^17^,^19^. While each model is consistent with observations in some species, it is inconsistent with others^3^.

A possible explanation for this diversity is that different ecological conditions, in different species, favour different signalling systems^8^,^22^,^23^,^24^,^25^. When food is relatively plentiful, parents can be selected to preferentially feed the offspring signalling the greatest need^8^. In contrast, when food is scarce, parents can be predicted to ignore begging and preferentially feed bigger chicks^8^. Consequently, a single factor—the extent to which parents can acquire enough food to feed all their offspring—could lead to the stability of different signalling systems, and hence explain the extreme variation across species in who begs and how parents respond to begging (Fig. 2)^3^,^8^,^25^.

There has, however, been no empirical test of whether different signalling systems have evolved in response to differences in relative food availability. While many studies have been conducted within species, it is hard to generalize their findings because of differences in environmental context and life history variables between species. We therefore conducted a comparative study to test whether signalling and provisioning correlate with the likelihood that parents can acquire enough food to rear a complete brood. We collected data on 143 bird species, examining how offspring signalling relates to their condition, and how parents respond to signals and cues of offspring condition. Our aim was to test the hypothesis that variation in food availability can explain variation in offspring communication and parental provisioning strategies.

We examined two possible determinants of parents' ability to acquire enough food to rear all their offspring: environmental predictability and environmental quality^8^,^18^,^26^,^27^,^28^. First, in species where environmental conditions are more predictable, parents are more likely to produce the clutch size that leads to all of their offspring surviving^27^,^28^,^29^. Conversely, in unpredictable environments, parents may lay an optimistic number of eggs, and are only be able to rear all their offspring in particularly good years^18^,^19^,^27^,^28^,^29^. Such species often begin egg incubation before they have completed a clutch, creating a size hierarchy: when conditions are worse, only the biggest and best quality chicks survive to fledge^18^,^27^,^28^,^29^. We classified species as ‘brood reducing' if hatching is asynchronous or if the later-hatched offspring die at greater rate, as is expected with low environmental predictability^18^,^27^,^28^,^29^. If this information was not available, we used a conservative cutoff of 75% broods in the population typically experiencing the starvation of at least one chick. We classed all other species as having a ‘whole-brood survival' strategy, as is expected with relatively high environmental predictability.

Our second determinant of food availability was current environmental quality^8^. In unusually good conditions, parents are more likely to be able to acquire enough food to feed all their offspring, even if they are typically brood reducing. We categorized environmental quality as good, average or poor compared with the norm for that population, dependent upon ecological measures or experimental manipulations. For example, owls experiencing a crash in the vole population^30^ are in a poor environment, and pigeons supplemented with mealworms and grain^31^ are experiencing a good environment.

We found that the strategies of both offspring and parents depend upon environmental predictability and quality. In relatively stable and unusually good environments, offspring signal their need and parents distribute food according to begging. In contrast, in relatively unpredictable and unusually poor environments, offspring signal their quality and parents are more likely to feed offspring based on signals or cues of quality. These results allow us to explain why opposite patterns have been observed in different species, with parents preferentially feeding offspring in either worse or better condition.

## Results and Discussion

### Offspring signalling strategies

**Figure 3:**
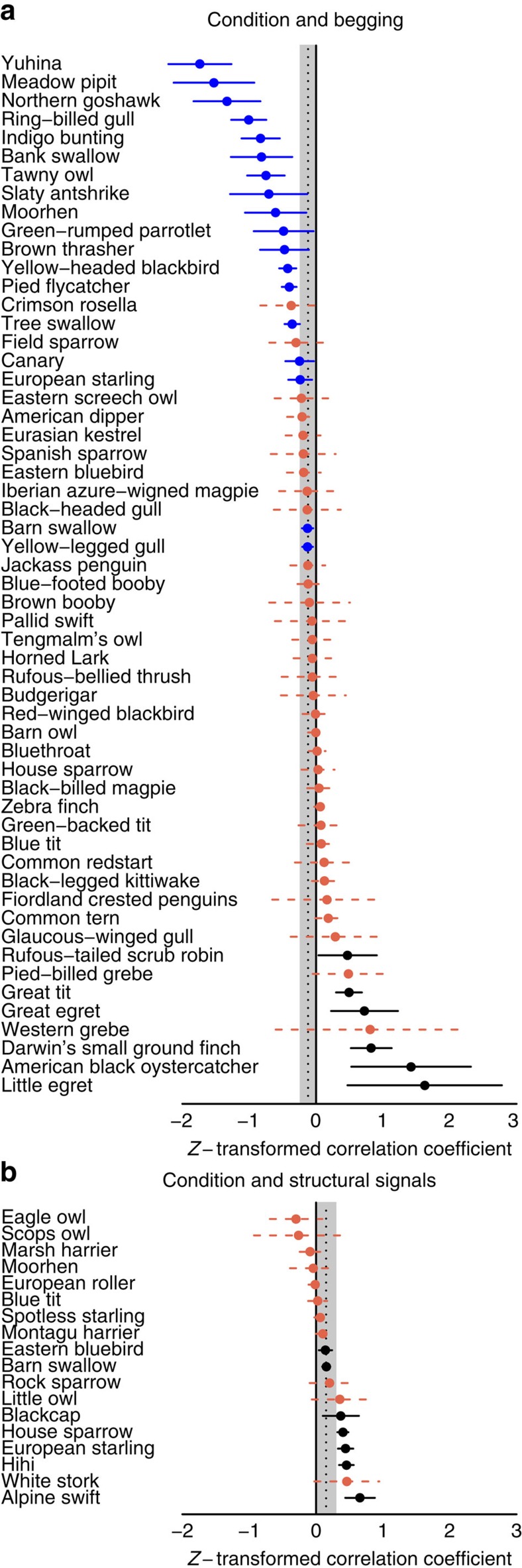
Variation in offspring signals across species. Circles represent species' mean *z*-transformed correlation coefficient between condition and (**a**) begging intensity (*N*=56 species) and (**b**) structural signal intensity (*N*=18 species). The grand mean and 95% credible interval (CI) are denoted by the shaded bar. Blue, solid lines indicate that chicks that in worse condition signal more. Black, solid lines indicate that chicks in better condition signal more. Red, dashed lines indicate no effect of condition on signals. Lines show 95% CI (±s.e. × *t*_critical_). s.e. was estimated from the pooled number of broods across all studies.

To determine what information is encoded in chick signals, we calculated the correlation coefficient (effect size) between offspring long-term condition and (1) begging and (2) structural signals (Fig. 3). The coefficient varies between ±1, with positive values meaning that chicks in better condition beg more or have larger structural signals, and negative values implying that chicks in worse condition beg more or have larger structural signals. Long-term condition, or the likelihood that offspring will survive to adulthood and reproduce, is information that obviously influences parents' fitness^3^,^6^,^17^,^32^. Long-term condition was captured by health, body condition, changes to food intake over multiple days, weight and rank within the brood. These different measures reflect factors that parents may or may not be able to assess directly to different degrees^3^,^4^,^14^.

Our first prediction was that chicks in worse condition should be more likely to beg, or beg more intensely, in better environmental contexts and in species that generally rear the whole brood. Under these conditions, all offspring will be more likely to survive to maturity, and hence honest signalling of need can be favoured. We examined vocal begging and posture, as birds could adjust these behaviours flexibly in response to need.

**Figure 4:**
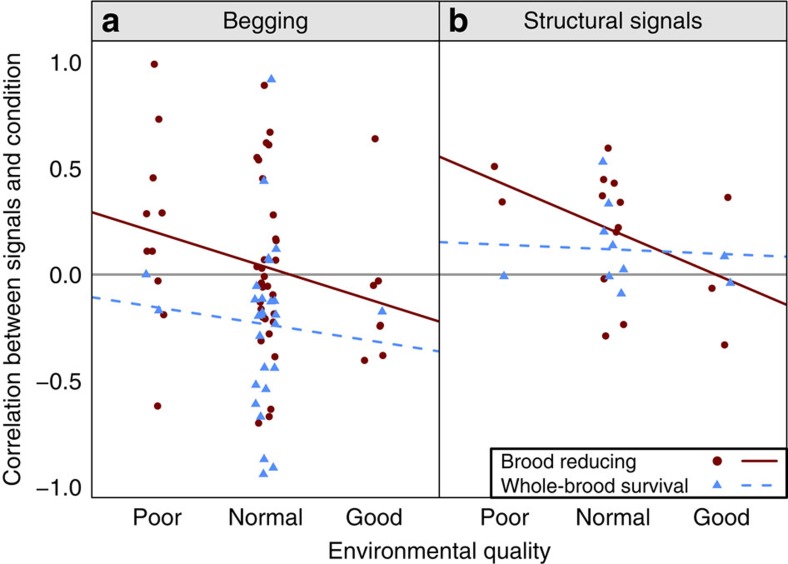
Brood reduction likelihood determines whether low or high condition chicks signal more. Data points represent each species' mean correlation coefficient (effect size) of offspring condition on signal intensity in that environment. Positive correlations indicate chicks in better condition signal at a higher intensity, and negative correlations indicate chicks in worse condition signal more. This is a graphical simplification; analyses were run on the full data set per effect size reported, not species' means. (**a**) Chicks in worse condition were more likely to beg the most in good environments (MCMCglmm, pMCMC=0.001), and in species which tend to raise the whole brood (MCMCglmm, pMCMC=0.02, *N*=56 species). This represents the scenario where brood reduction is least likely. (**b**) Chicks in better condition were more likely produce more intense structural signals in brood-reducing species in poor environments (MCMCglmm, pMCMC=0.003, *N*=18 species). This represents the scenario where brood reduction is most likely.

As predicted, we found that chicks in worse condition were more likely to beg in species that rear a complete brood (phylogeny-based, Bayesian generalized linear mixed models with Markov chain Monte Carlo methods: pMCMC=0.001; Table 1a; Fig. 4a). In contrast, in brood-reducing species, there is no general correlation between chick long-term condition and begging intensity (pMCMC=1; Table 1a). Furthermore, across all species, chicks in worse condition were more likely to beg more intensely in better environments (pMCMC=0.001; Table 1a). None of our results were influenced by the measure of condition or begging used in the original studies (Supplementary Tables 1–2). These results are consistent with a greater likelihood of signalling of need in species trying to rear all their offspring.

We then examined a mode of parent–offspring communication that we predicted could function as a signal of quality: structures such as mouth colour, ultraviolet reflectance and mouth size^22^,^33^,^34^. These require a relatively long-term investment of resources such as carotenoids, and so are more likely to reflect long-term quality rather than short-term need^33^. Consequently, these structural signals are more likely to be used as signals of quality, and so we predict that they will be greater in species living in unpredictable and poor environments, where only a fraction of the brood will be reared.

As predicted, we found that chicks in better condition tended to produce more intense structural signals in brood-reducing species (pMCMC=0.02), but not in species that rear the whole brood (pMCMC=0.4; Table 1b; Fig. 4b). This is consistent with structural signals being used to signal quality when brood reduction is possible. Furthermore, there was a significant interaction between brood reduction strategy and environmental quality in the predicted direction (pMCMC=0.003; Table 1b). Specifically, brood-reducing species showed an increased tendency for better condition chicks to produce greater structural signals in poorer environments, which is when brood reduction is most likely. These results are consistent with a decrease in relative food availability selecting for chicks to signal quality to their parents, to avoid being the chick left to starve.

Our analyses support our hypothesis that when raising a complete brood is likely, selection should favour chicks that signal need through behavioural begging. In contrast, when parents are unlikely to raise a complete brood, selection appears to favour chicks that signal quality through structural signals. However, caution is required when interpreting these results, because signalling theory predicts that signals must transmit information about a cryptic aspect of quality that parents cannot otherwise detect^4^,^15^,^20^. While researchers may have captured this with measures such as immunocompetence^33^,^35^, measures such as body mass may be less likely to reflect some cryptic aspect of condition. A stronger approach to test our hypothesis would be to examine parental response to begging signals, structural signals and body size cues, in terms of how parents distribute food among offspring

### Parental feeding strategies

**Figure 5:**
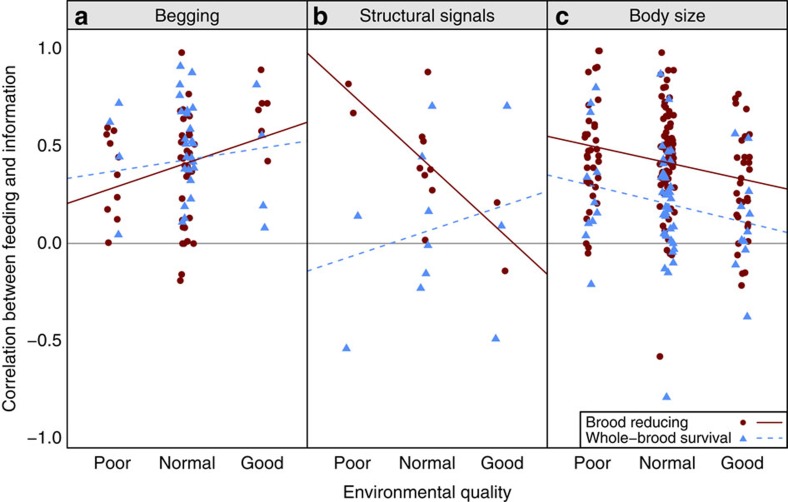
Brood reduction likelihood determines how parents respond to chick signals and cues. Data points represent species' mean correlation coefficient (effect size) of signal intensity or body size on food allocation in that environment. Positive correlations indicate larger chicks or those signalling more intensely receive more food. This is a graphical simplification; analyses were run on the full data set per effect size reported, not species' means. (**a**) Parents respond more to begging as the environment improves (MCMCglmm, pMCMC=0.01, *N*=61 species). (**b**) Brood-reducing species paid more attention to structural signals in poorer environments, whereas species that rear the whole brood show a consistently low response, though low sample size keeps this interaction non-significant (MCMCglmm, pMCMC=0.09, *N*=15 species). (**c**) Parents showed a stronger preference for larger chicks in poorer environments (MCMCglmm, pMCMC=0.006) and brood-reducing species (MCMCglmm, pMCMC<0.0002 and *N*=120 species).

To determine what information parents use when allocating food, we estimated the strength of the correlation between feeding and three sources of information about chick condition: begging, structural signals and body size cues (Fig. 5). Parents may respond to all, none or a combination of these signals and cues when allocating food. As far as possible, we included only data that isolated the individual effects of each of these information sources; for example, begging height, which combines both body size and begging posture, was excluded. The direction of each correlation coefficient was based solely on whether chicks signalling more were fed more, and our analyses of parental response made no assumptions about what information was included in the signal or cue.

**Figure 6:**
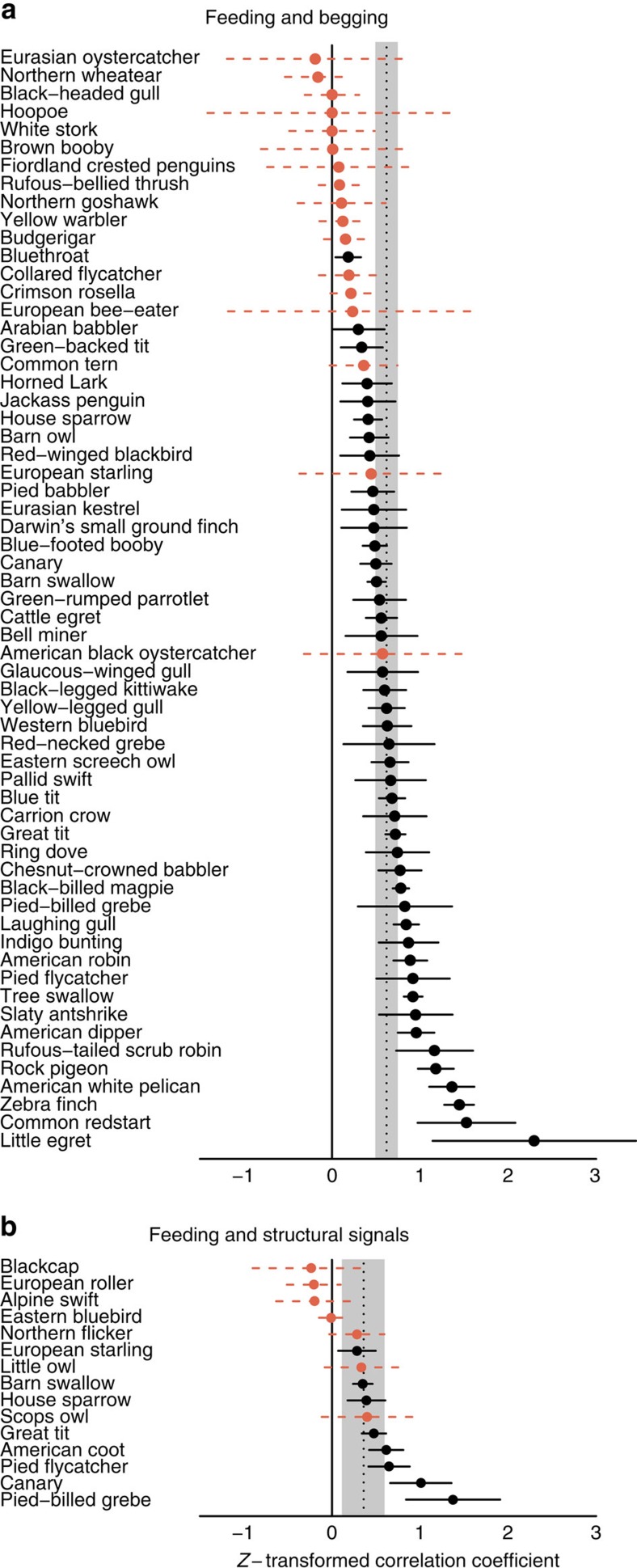
Variation in parental responsiveness to offspring signals across species. Circles represent species' mean *z*-transformed correlation coefficient between feeding and (**a**) begging (*N*=61 species), and (**b**) structural signals (*N*=15 species). The grand mean and 95% credible interval (CI) are denoted by the shaded bar. Black, solid lines indicate chicks that signal more are preferentially fed more. Red, dashed lines indicate no effect of signals on feeding. Lines show 95% CI (±s.e. × *t*_critical_). s.e. was estimated from the pooled number of broods across all studies.

**Figure 7:**
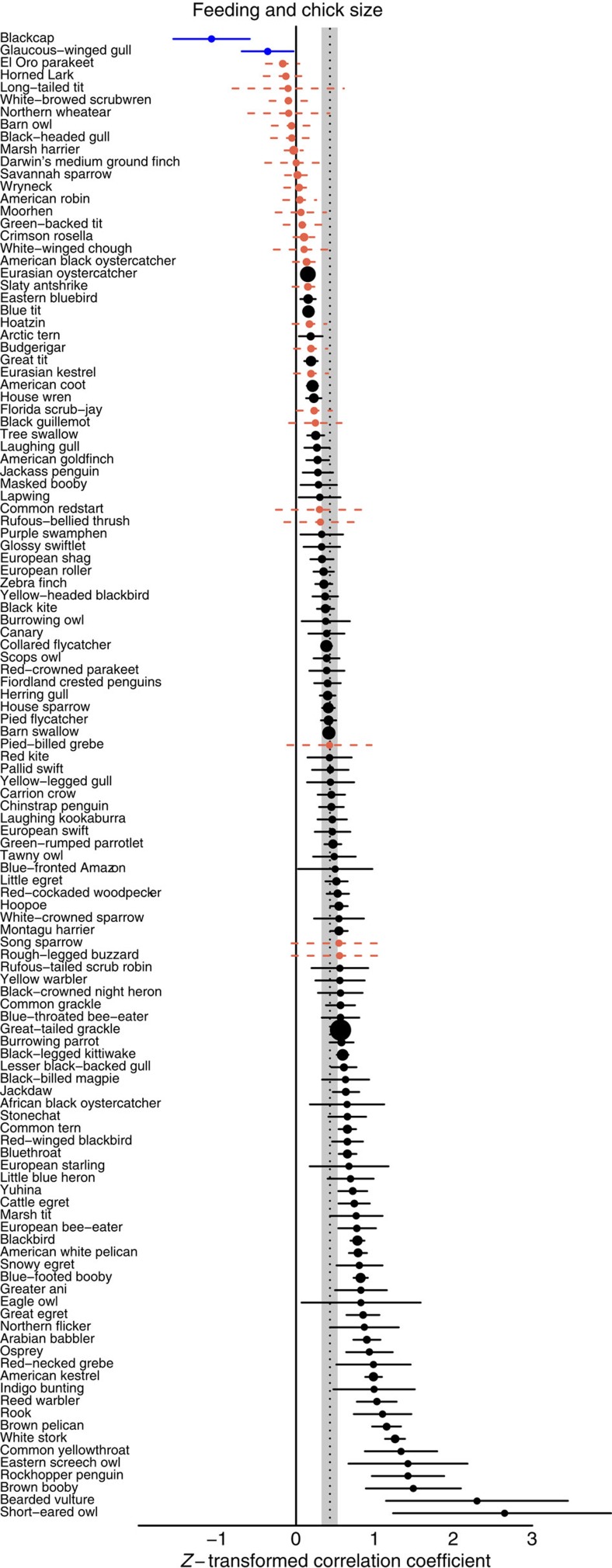
Variation in parental responsiveness to offspring size across species. Circles represent species' mean *z*-transformed correlation coefficient between feeding and chick body size (*N*=120 species). The grand mean and 95% credible interval (CI) are denoted by the shaded bar. Black, solid lines indicate chicks that signal more are preferentially fed more. Red, dashed lines indicate no effect of signals on feeding. Lines show 95% CI (±s.e. × *t*_critical_). s.e. was estimated from the pooled number of broods across all studies.

On average, parents preferentially feed chicks that beg more (pMCMC<0.0001; Table 2a), have brighter and more saturated mouths (pMCMC=0.009; Table 2b), and are larger (pMCMC<0.0002; Table 2c). The responsiveness to begging varied across species, with parents not preferentially feeding the chicks that beg the most in 17 of the 61 species studied (Fig. 6a; total heterogeneity (*I*^2^), the proportion of observed variance that reflects true differences in correlation coefficients: 23.2%; Supplementary Table 6). Responsiveness to structural signals was also variable across species, with no effect of signal intensity on feeding in almost half of species studied (*I*^2^=16.9%; Fig. 6b; Supplementary Table 6). Surprisingly, given the common assumption that parents want to feed the neediest offspring, parents almost universally prefer feeding larger offspring: only 2 of 120 species feed smaller chicks more (*I*^2^=15.4%; Fig. 7; Supplementary Table 6). None of our results were influenced by the measure of feeding preference used in the original studies (Supplementary Tables 3–5).

We predict that parents will preferentially feed chicks in the greatest need when there is a relatively high likelihood that parents will have enough food to rear a complete brood. Given that that chicks signal need behaviourally with vocal calls and posturing (Fig. 4a), we expect that parents should be more likely to respond to such begging in relatively good environments. In contrast, when parents are unlikely to have enough food to rear a complete brood, we predict that parents should prefer to feed the better quality chicks, irrespective of begging intensity. Quality could be assessed by a cue such as body size or by chicks signalling quality with structures (Fig. 4b). In this context, we expect a positive correlation between food allocation and offspring size and/or structural signals.

As predicted, we found that parents preferentially fed the chicks that begged the most in good environments, but were less responsive to begging in poor environments (pMCMC=0.01; Table 2a; Fig. 5a). This pattern did not differ depending upon whether a species was brood reducing or rears the whole brood (pMCMC=0.5; Table 2a).

In contrast, we expect the opposite pattern with structural signals of quality and body size: when parents are unlikely to have enough food to rear all of their offspring, we predict they will respond more to structural signals and body size. As predicted, we found that parents preferentially fed chicks with more colourful mouths and/or larger bodies in poor environments, and responded less to structural signals and body size cues in good environments (structural signals: pMCMC=0.007; size: pMCMC=0.006; Table 2b,c; Fig. 5b,c). Furthermore, the preference for larger chicks was greater in brood-reducing species than in species where the whole brood survives (pMCMC<0.0002; Table 2c). Parents in brood-reducing species were also more likely to feed chicks based on structural signals, although not significantly, perhaps because of low sample size for this comparison (pMCMC=0.09, *n*=6 whole-brood survival species, 9 brood-reducing species; Table 2b).

Overall, a clear pattern emerges: the probability of successfully raising all offspring from a nest determines the system of communication between parents and their offspring across species. In predictable and/or unusually good environments, offspring in worse condition are more likely to beg (Fig. 4a), and parents are more likely to feed individuals begging at a higher rate (Fig. 5a). These results are predicted by signal of need models, where parents expect to rear a complete brood^3^,^14^,^15^,^16^. In contrast, in unpredictable and/or poor environments, offspring in better condition have more intense structural signals (Fig. 4b), and parents are more likely to feed chicks that are larger or have more intense structural signals (Fig. 7b,c). These results are predicted by signal of quality models, where parents rear only a fraction of their offspring, or by models where signalling is not stable, and parents just respond to cues of quality^3^,^8^,^21^,^36^,^37^. Another possibility is that parents respond less to variation in begging when food availability is low simply because all chicks are hungry, and therefore beg at similar rates. Irrespective of whether begging provides less information or parents are selected to ignore it, the outcome is the same: begging becomes a less useful signal in worse environments, while body size and structural signals become more important.

Our study relied on the fact that there was sufficient variation across species in food availability to produce different evolutionary outcomes. In some cases, there may even be sufficient environmental variation within a species that individuals will be selected to adjust their behaviour conditionally in response to local conditions. For example, hihi parents become less sensitive to their offspring's mouth colour when they are supplemented with additional food^38^. Similarly, alpine swifts who breed early in the season, when food availability is greater, prefer nestlings with lower ultraviolet reflectance, while parents who breed later, under worse conditions, switch to preferring nestlings with greater reflectance^39^.

## Conclusions

More generally, one of the major outstanding challenges for our understanding of how communication evolves is to explain why species have such diverse communication systems. We have shown how variation in environmental quality can explain differences in communication between offspring and their parents. In relatively good environments, there is less conflict, and offspring can be selected to signal need to their parents. In contrast, in relatively poor environments, there is more conflict, and parents are expected to respond to quality rather than need. Furthermore, this can occur via either offspring signalling quality or parents ignoring signals and instead relying on cues such as body size. This variation is why hundreds of empirical studies on begging and parental response have not yet led to a consensus on exactly what information is transmitted through offspring signals, or how parents respond to various signals^3^,^4^,^5^,^6^,^7^. Our results suggest that this variation reflects different communication systems being stable in different species.

## Methods

### Data collection

We conducted a literature search on Web of Science and Google Scholar using the keywords ‘beg', ‘parent–offspring', ‘bird', ‘begging', ‘communication' and ‘provision' (see Supplementary Fig. 1 for PRISMA flowchart detailing data collection). We performed backwards and forwards citation searches on all studies. We included studies published before August 2014, as well as unpublished data sets from five researchers. We included all papers with any measure relating to the relationship between chick long-term condition and (1) behavioural begging or (2) structural signals, and food allocation and (3) behavioural begging, (4) structural signals or (5) size cues (see Supplementary Table 9 for a list of excluded of studies). We excluded studies if it was impossible to determine whether parents were responding to begging or to size cues. We excluded effect sizes where the only measure of chick condition was proximate hunger rather than long-term condition. We only included effect sizes for the relationship of begging on within-brood food allocation, rather than on increases in overall parental feeding effort, as these represent fundamentally different aspects of parental care. We excluded data on species that lay only one egg per brood, as selective pressures on these offspring are likely to differ from species laying multiple eggs per brood. If relevant data were given in papers without statistical tests, such as raw means and s.e.'s, we estimated effect sizes. This resulted in a data set of 1,544 effect sizes (correlation coefficients) from 306 studies on 143 species (Supplementary Data 1). The data set contains a diverse range of species, spanning 51 families in 19 different orders.

### Measures of offspring condition

We examined the effect of long-term condition on signalling intensity^32^. Our proxies for long-term condition were health (for example, experimental immune challenge, parasite load, carotenoid supplementation), body condition (for example, body mass to skeletal size ratio, blood glucose levels), weight (for example, body mass), rank within the brood (for example, hatching rank, dominance rank, body mass or skeletal size rank) and experimental manipulations that affected food intake over multiple days (for example, experimentally reduced or enlarged broods, with the assumption that chicks in larger broods receive less food per capita).

We excluded studies that examined only the effect of short-term food deprivation, that is, hunger. While hunger and condition may not be truly separable, they represent very different selection pressures^3^,^4^,^5^,^6^,^17^,^32^,^40^. For example, although each piece of food eaten contributes to the likelihood that a chick will survive, the fitness benefit of food to fatally diseased chicks is zero, because they will not live to breed^3^,^34^,^40^. Furthermore, the influence of hunger on begging is already well established^2^,^3^. Consequently, we focused on the influence of long-term condition, and so data on the relationship between hunger and signal intensity were not included in analyses of offspring strategies. For example, we excluded data such as Kilner^34^ finding that canary chicks' mouths get redder as they become hungrier over 40 min. However, our analyses of parental behaviour made no assumptions about what information was transmitted by signals. Therefore, feeding in response to mouth redness would be included in analyses on parental behaviour, just as the response to begging calls, which may be influenced by both hunger and condition, was included. It would be an interesting task for the future to examine whether and how hunger interacts with measures of long-term condition^4^.

### Measures of signalling and provisioning

Many aspects of the behavioural and structural signalling suite were reported in the literature, such as begging amplitude, duration, latency, likelihood, call structure, posture, ultraviolet reflectance of the gape or flange, carotenoid saturation of the gape or flange, or colouration of specialized skin patches and feathers only present during the nestling period. Different measures of food allocation were also reported, such as weight gain over a short time period, actual food intake, number of food items received, likelihood of being fed, growth rate and mortality. We assumed that all measures of signal intensity and food allocation were driving towards the same biological phenomenon, and so included all reported statistics in our analyses. Parents' responses to begging signals, structural signals and body size cues were analysed separately. Because measures of feeding preference such as mortality could have been partially confounded by how we classified environmental predictability, we tested whether the measure of feeding affected the strength of the correlation coefficient, but found no difference between any of the proxies for feeding preference (pMCMC>0.05, see Supplementary Tables 3–5). Because test statistics were converted to a standardized scale, differences between the various measures of begging intensity or feeding preferences should not influence the overall trends seen. Study methodology, such as which measure of long-term condition was reported or whether the study was experimental or observational, had no impact on effect size (pMCMC>0.05 in all cases, see Supplementary Tables 3–5).

### Data on brood reduction strategy

We classified species as brood reducing if hatching is asynchronous (24 h or more passes between the hatching of the first and last chick in the brood) and if nestling mortality follows a stereotypical pattern of later-hatched nestlings dying at a greater rate due to starvation, siblicide or infanticide^27^. If that data were not available, we assessed whether partial brood mortality is typical (at least one chick starves in at least 75% of broods in the population)^18^,^27^,^28^. Many brood-reducing species experience lower rates of starvation^3^, but this conservative criterion allows us to identify species with a very clear strategy of brood reduction based on environmental factors, rather than incidental starvation. The combination of hatching and mortality patterns allowed us to distinguish between species employing a true brood reduction strategy and those with asynchronous hatching for other reasons, such as spreading offspring demand evenly over the nestling period or avoiding chilling earlier-laid eggs^28^.

### Data on environmental quality

To evaluate how environmental conditions interact with life history traits across species, we categorized populations as experiencing normal, better than normal or worse than normal environments, based on experimental manipulations (parents were fed reduced or supplemented diets or chick demand was artificially increased or decreased), ecological measures (such as prey density, date or rainfall), or average mortality across different years in long-term observational studies. Only long-term manipulations of food availability over multiple days were included, to ensure chick condition, and not simply hunger, was affected by the ecological variation. If no information on environmental quality was available, studies were conservatively classified as normal conditions.

### Statistical analyses

To evaluate the strength of the relationships across studies and species, we transformed any test statistic measuring either an effect of long-term condition on signal intensity, or an effect of chick signals or cues on feeding into a standardized effect size (Fisher's *Z*-transformed correlation coefficient)^41^,^42^,^43^,^44^. These correlation coefficients follow a normal distribution, account for different scales in their original measurements, are well suited to the ordered nature of the data and are more straightforward to interpret than standardized difference in means^41^. Before analyses, we decided not to exclude potential outliers. Fisher's *Z*-transformed correlation coefficients were analysed using the MCMCglmm package in R, which implements Bayesian generalized linear mixed models with Markov chain Monte Carlo methods^45^,^46^. Models were weighted by sample size and controlled for phylogeny and repeated measures on the same study and species. Sample size was determined as the number of broods used to generate the original test statistic, because this is a standard measure across studies. It also conservatively avoids pseudoreplication if chick number or number of observations were used as the sample size. Environmental quality was treated as a three-level ordered categorical variable, and brood reduction strategy as a two-level categorical factor. We obtained phylogenies from Birdtree.org, and models were run on 100 random phylogenetic trees with Ericson and Hackett backbones, and then averaged^47^. Analyses were run separately for each relationship.

Forest plots and species-level analyses were conducted with the metafor package in R^45^,^48^. We assessed the heterogeneity of our data using *I*^2^, which is a descriptive measure of the proportion of observed variance that reflects true differences in correlation coefficients^41^,^49^,^50^. Results related to heterogeneity and random effects can be found in Supplementary Tables 6–7. Example R code can be found in Supplementary Note 1 or requested from authors. We used ASReml analyses to confirm the results of our meta-analysis (Supplementary Methods; Supplementary Table 8)^51^,^52^,^53^.

### Tests for publication bias

Although we did not expect to find one true effect size across all studies and species^41^, we tested our meta-analysis for publication bias using the regression test for funnel plot asymmetry (Egger's test) in the ‘metafor' package in R^48^. We calculated the average effect size per study and compared it with its variance to determine whether studies with smaller sample sizes were more likely to show extreme effects. We found no evidence of publication bias in the relationships between: (1) offspring condition and begging (*z*=0.54, *P*=0.59); (2) offspring condition and structural signals (*z*=0.93, *P*=0.35); (3) feeding and begging (*z*=−0.49, *P*=0.63); (4) feeding and structural signals (*z*=0.91, *P*=0.36); and (5) feeding and body size cues (*z*=−1.59, *P*=0.11).

### Tests for confounding methodological factors

We recorded additional information on study methodology for each effect size, including the following: the sample size (number of broods) of that measurement; the type of begging variable (three-level factor: whether or not begging occurred; any continuous intensity measure (for example, duration, amplitude and posture); hunger treatment: experimentally deprived or satiated, with the assumption that hungry chicks beg more); the type of feeding variable (four-level factor: whether or not feeding occurred; any continuous measure of feeding (feeding rate or weight of food received); growth of mass or body structures (tarsus and primary feather), either rate or final size attained, with the assumption that growth rates reflect feeding rates at least in part; and mortality risk before fledging, with the assumption that mortality rates reflect feeding rates at least in part. Nestlings typically died of starvation. Predation risk was excluded as much as possible by considering only partial brood losses); the type of long-term condition variable (five-level factor: health, rank, weight, condition and long-term changes to food intake); whether the offspring contrast was dichotomous (bigger versus smaller) or continuous (all offspring included); whether the female, male, both sexes combined or a helper was the responder (four-level factor); and whether the data were experimental or observational (two-level factor). Not all study methodology variables were relevant for all aspects of this communication system. Analyses of potential confounding factors were only run if at least two factor levels had at least 10 effect sizes.

No methodological factors had an impact on any of the five relationships tested in this communication system (MCMCglmm phylogenetically controlled and weighted regression: pMCMC>0.05). The 95% credible intervals of the correlation coefficients for each methodological factor are presented in Supplementary Tables 1–5.

Because measures of feeding preference such as mortality and growth rate may be partially confounded by how we classified environmental predictability, we ran models excluding all effect sizes generated using these measures. There was no change in the significance of results for any parental responsiveness model, despite a reduction in sample size of 50 species. In the model of how much parents feed their offspring in response to size cues, the slope for environmental quality changed from −0.14 (pMCMC<0.0002) to −0.25 (pMCMC=0.005), and the difference between brood reducing and whole-brood survival changed from −0.30 (pMCMC=0.006) to −0.26 (pMCMC=0.018). In the model of how much parents feed their offspring in response to begging, the slope for environmental quality changed from 0.35 (pMCMC=0.01) to 0.34 (pMCMC=0.01). In the model of how much parents feed their offspring in response to structural signals, the slope for environmental quality did not change, the difference between brood reducing and whole-brood survival changed from −0.45 (pMCMC=0.09) to −0.46 (pMCMC=0.06), and the interaction from 0.61 (pMCMC=0.09) to 0.58 (pMCMC=0.10).

### Heterogeneity

We measured the heterogeneity in our data set with *I*^2^, the proportion of observed variance due to true differences in effect sizes, rather than measurement errors (Supplementary Table 6)^41^,^49^,^50^. Total *I*^2^ was calculated by dividing the summed variance attributed to phylogeny, species, study and units by the overall variance observed in the data (variance attributed to measurement error, phylogeny, species, study and units). Higher values of *I*^2^ indicate that more of the observed variance is true rather than due to measurement error, with 25%, 50% and 75% as low, moderate and high benchmarks, respectively^41^,^49^.

*I*^2^ describes the amount of true heterogeneity seen, but these results should be interpreted with caution. *I*^2^ is independent of the absolute value of the variance observed, which is good because it does not vary based on the scale or number of studies included in the meta-analysis^41^. However, this measure does not take into account the dispersion of effect sizes, only the precision with which effect sizes were measured^5^. Thus, identical *I*^2^ values could be obtained even if the between-species variance differed by an order of magnitude^41^. If the true effect size for each species or study is spread over a wide range, *I*^2^ cannot capture this dispersion.

## Additional information

**How to cite this article:** Caro, S. M. *et al*. Unpredictable environments lead to the evolution of parental neglect in birds. *Nat. Commun.* 7:10985 doi: 10.1038/ncomms10985 (2016).

## Supplementary Material

Supplementary Information Supplementary Figure 1, Supplementary Tables 1-9, Supplementary Note 1, Supplementary Methods and Supplementary References.

Supplementary Data 1 Full data set used for meta-analyses, including citations and references for brood reduction strategy.

## Figures and Tables

**Figure 1:**
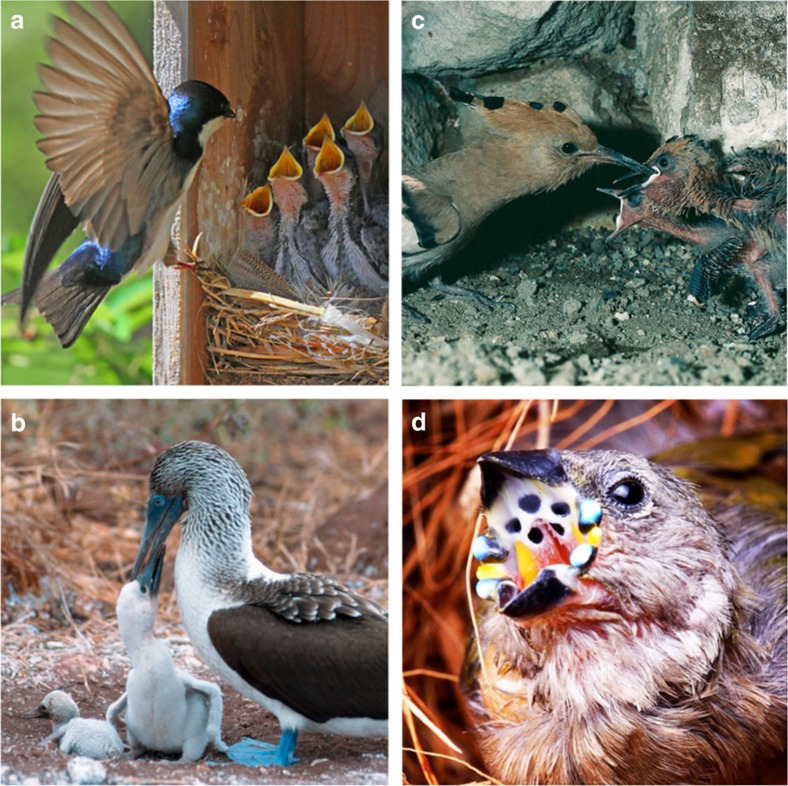
Variation in parental provisioning. In every species of bird with parental care, chicks appear to have evolved signals designed to maximize their chance of being fed, such as vocalizations, begging postures and bright mouths. However, the way parents respond to information about their offspring differs markedly across species. Tree swallows *Tachycineta bicolor* feed the chick begging the most (**a**). Others sometimes neglect begging offspring, such as the blue-footed booby *Sula nebouxii* (**b**) and the hoopoe *Upupa epops* (**c**) which instead preferentially feed larger chicks. Gouldian finch *Erythrura gouldiae* parents (**d**) may preferentially feed offspring with elaborate structural ornaments around their mouths. (Photos courtesy of (**a**) M. Sodicoff. (**b**) This figure is not covered by the CC BY licence ©Damschen/ARCO/naturepl.com. All rights reserved, used with permission. (**c**) This figure is not covered by the CC BY licence © L.M.R. Gordón. All rights reserved, used with permission; and (**d**) This figure is not covered by the CC BY licence (**c**) G. Grall, National Aquarium, Baltimore. All rights reserved, used with permission.

**Figure 2:**
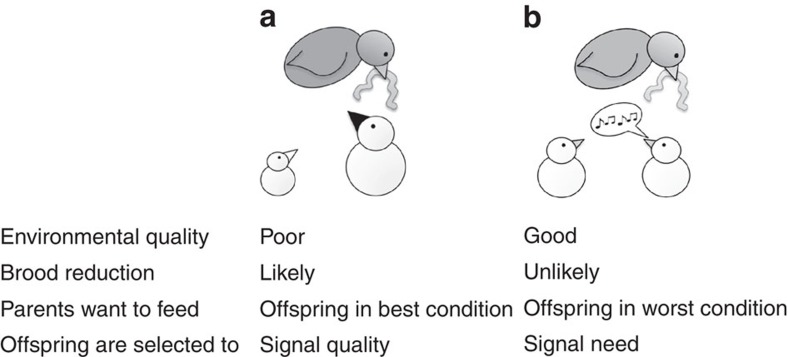
Ecological variation and diversity in signalling systems. (**a**) In unpredictable environments, parents may produce a larger brood than future environmental conditions will support. This selects for parents to preferentially feed the offspring with the highest chance of survival. We predict parents will assess quality by cues such as body size, or structural signals such as dark mouths. Offspring may still beg, but parents should ignore begging in favour of other information. (**b**) In contrast, in predictable environments, parents will lay an appropriate number of eggs and food will be plentiful enough to rear all their offspring. Here parents will be selected to preferentially feed offspring in the worst condition. We predict offspring should signal need through begging, and parents will feed those begging more.

**Table 1:** Environmental and life history influences on offspring signalling strategies.

	Posterior mean *Z*	95% Credible interval	pMCMC
*(a) Begging*			
Brood reducing	0.01	−0.19 to 0.17	0.95
Whole-brood survival	−0.30	−0.47 to −0.14	0.001***
Reduction difference	−0.37	−0.66 to −0.05	0.02*
Environment	−0.29	−0.50 to −0.07	0.001**
Reduction × environment	−0.07	−0.33 to 0.22	0.62
Grand mean	−0.12	−0.26 to 0.01	0.09.
			
*(b) Structural signals*			
Brood reducing	0.31	0.08 to 0.57	0.02*
Whole-brood survival	0.10	−0.13 to 0.36	0.4
Reduction difference	−0.21	−0.47 to 0.09	0.18
Environment	−0.53	−0.77 to −0.28	0.0001***
Reduction × environment	0.49	0.16 to 0.80	0.003**
Grand mean	0.15	−0.00 to 0.32	0.06.

Results of MCMCglmm analyses on Fisher's *Z*-transformed correlation coefficients (manuscript and figures report correlation coefficients). *p*<0.10, *p<0.05, **p<0.01, ***p<0.001.

(a) Behavioural begging: *n*=56 species, 96 studies, 247 effect sizes.

(b) Structural signals: *n*=18 species, 33 studies, 140 effect sizes.

**Table 2:** Environmental and life history influences on parental response strategies.

	Posterior mean *Z*	95% Credible interval	pMCMC
*(a) Begging*			
Brood reducing	0.70	0.52 to 0.89	0.0004***
Whole-brood survival	0.61	0.38 to 0.87	0.0002***
Reduction difference	−0.11	−0.40 to 0.16	0.5
Environment	0.35	0.05 to 0.61	0.01*
Reduction × environment	−0.16	−0.67 to 0.33	0.5
Grand mean	0.62	0.48 to 0.76	<0.0001***
			
*(b) Structural signals*			
Brood reducing	0.79	0.34 to 1.2	0.0006***
Whole-brood survival	0.34	−0.09 to 0.80	0.12
Reduction difference	−0.45	−0.97 to 0.08	0.09.
Environment	−0.71	1.22 to −0.20	0.007**
Reduction × environment	0.70	−0.10 to 1.50	0.09.
Grand mean	0.36	0.10 to 0.61	0.009**
			
*(c) Body size*			
Brood reducing	0.50	0.40 to 0.59	<0.0002***
Whole-brood survival	0.19	0.07 to 0.32	0.003**
Reduction difference	−0.30	−0.43 to −0.17	<0.0002***
Environment	−0.14	−0.25 to −0.04	0.006**
Reduction × environment	0.001	−0.20 to 0.18	0.98
Grand mean	0.43	0.31 to 0.54	<0.0002***

Results of MCMCglmm analyses on Fisher's Z-transformed correlation coefficients. *p*<0.10, *p<0.05, **p<0.01, ***p<0.001.

(a) Behavioural begging: *n*=61 species, 92 studies, 301 effect sizes.

(b) Structural signals: *n*=15 species, 20 studies, 60 effect sizes.

(c) Body size: *n*=120 species, 218 studies, 795 effect sizes.
