# Supplementary material for: Unpredictable environments lead to the evolution of parental neglect in birds
Source: Nat Commun. 2016 Mar 29;7:10985. doi: 10.1038/ncomms10985 (PMC4820566; doi:10.1038/ncomms10985)
Supplement: Supplementary Information — Supplementary Figure 1, Supplementary Tables 1-9, Supplementary Note 1, Supplementary Methods and Supplementary References. [file ncomms10985-s1.pdf]

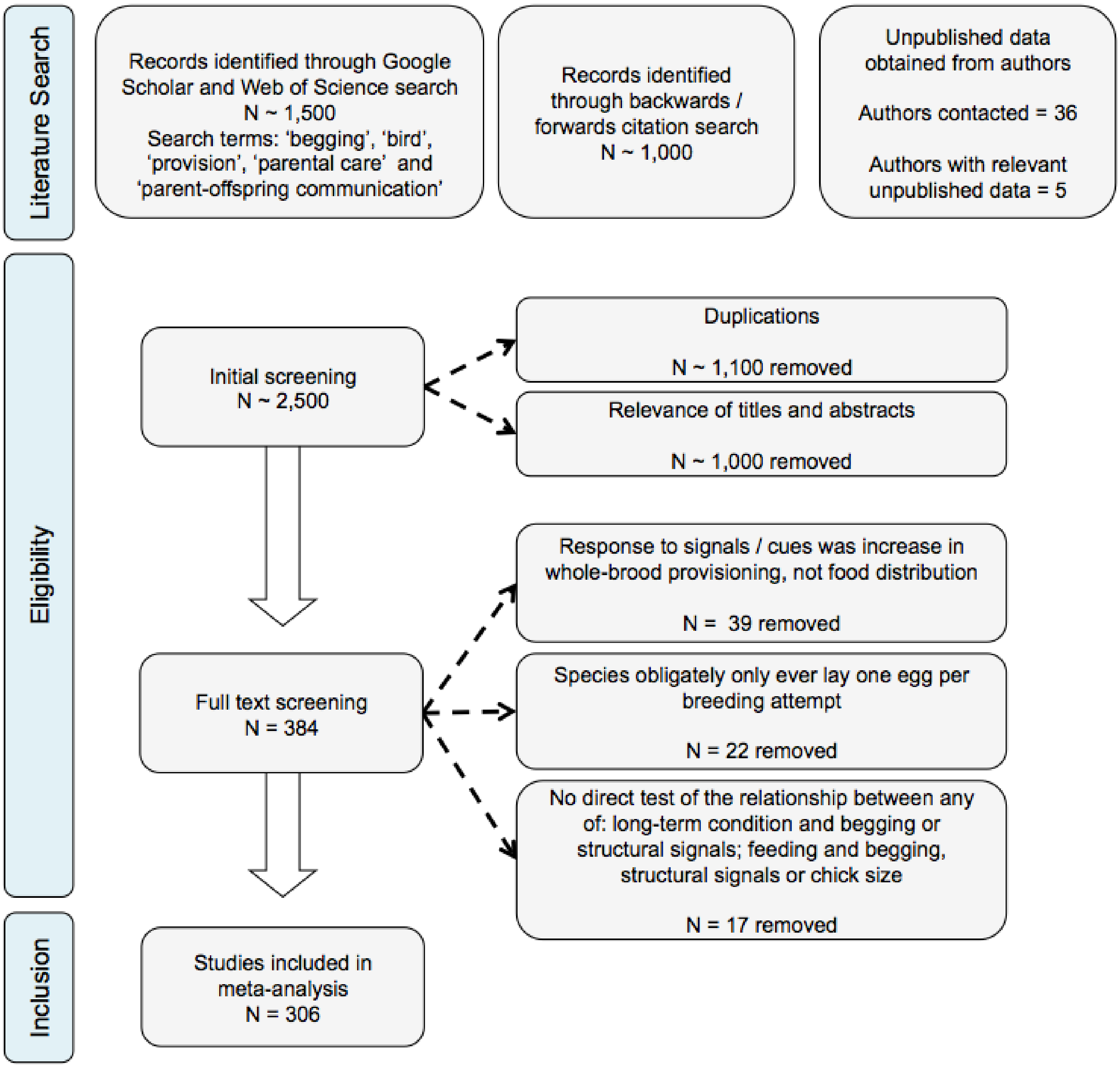

Supplementary Figure 1. PRISMA flow chart of search results and the study selection process. See Supplementary Table 9 for list of papers excluded from the analysis.

Supplementary Table 1. Condition and begging: tests for confounding methodological factors

		n	95% CI
**Study design**	Experimental	155	-0.29 to 0.00
	Observational	92	-0.36 to -0.05
**Long-term condition measure**	Long term change to food intake	47	-0.37 to -0.05
	Condition	31	-0.44 to -0.14
	Health	17	-0.47 to 0.08
	Size rank	117	-0.21 to 0.07
	Weight	35	-0.23 to 0.12
**How chick comparisons were made**	Continuous	180	-0.31 to -0.03
	Dichotomous	64	-0.36 to 0.02
	Whole brood	3	-0.55 to 0.14
**Beg variable type**	Continuous	226	-0.30 to -0.04
	Probability	21	-0.40 to 0.12
**Beg mode**	Audio	82	-0.32 to -0.01
	Posture	113	-0.41 to 0.00
	Combination	52	-0.31 to 0.01

N = number of effect sizes

Supplementary Table 2. Condition and structural signals: tests for confounding methodological factors

		n	95% CI
**Study design**	Experimental	100	-0.12 to 0.69
	Observational	40	-0.13 to 0.02
**Long-term condition measure**	Long term change to food intake	8	0.09 to 0.60
	Condition	48	-0.11 to 0.34
	Health	15	0.05 to 0.57
	Size rank	32	-0.04 to 0.44
	Weight	37	-0.08 to 0.39

N = number of effect sizes. Analyses not run: How chick comparisons were made: continuous (n = 133) vs dichotomous (n = 7)

Supplementary Table 3. Feeding and begging: tests for confounding methodological factors

		n	95% CI
**Study design**	Experimental	193	0.43 to 0.75
	Observational	108	0.47 to 0.84
**Feeding measurement type**	Food amount	113	0.36 to 0.73
	Chick growth	9	-0.11 to 0.78
	Probability	179	0.50 to 0.84
**How chick comparisons were made**	Continuous	237	0.48 to 0.78
	Dichotomous	4	-0.49 to 1.24
	High quality chicks	33	0.28 to 0.84
	Low quality chicks	27	0.27 to 0.86
**Beg variable type**	Continuous	240	0.49 to 0.79
	Hunger*	31	0.26 to 0.81
	Probability	30	0.17 to 0.82
**Beg mode**	Audio	51	0.24 to 0.73
	Posture	147	0.53 to 0.89
	Combination	103	0.38 to 0.74
**Which parent was tested**	Both	235	0.46 to 0.76
	Female only	36	0.33 to 0.82
	Male only	26	0.48 to 1.03
	Helper	4	-0.22 to 1.19

N = number of effect sizes
*Chicks were food deprived, and authors presumed this increased their behavioural begging intensity. Feeding rates were typically compared pre- and post-deprivation

Supplementary Table 4. Food allocation and structural signals: tests for confounding methodological factors

		n	95% CI
**Feeding measurement type**	Food amount	38	0.08 to 0.69
	Chick growth	10	-0.05 to 0.73
	Mortality	3	-0.21 to 0.90
	Probability	9	-0.14 to 0.86
**How chick comparisons were made**	Continuous	44	0.07 to 0.60
	High quality chicks	9	0.09 to 0.83
	Low quality chicks	7	0.07 to 0.83

N = number of effect sizes. Analyses not run: Study design: experimental (n = 59) vs observational (n = 1). (b) Which parent tested: both (n = 54), female only (n = 3) or male only (n = 3).

Supplementary Table 5. Food allocation and body size: tests for confounding methodological factors

		n	95% CI
**Study design**	Experimental	384	0.25 to 0.55
	Observational	430	0.31 to 0.60
**Feeding measurement type**	Food amount	282	0.22 to 0.53
	Chick growth	288	0.32 to 0.62
	Mortality	140	0.29 to 0.60
	Probability	104	0.24 to 0.61
**How chick comparisons were made**	Continuous	449	0.25 to 0.51
	Dichotomous	361	0.35 to 0.63
	Whole brood	4	0.15 to 1.65
**Which parent was tested**	Both	670	0.31 to 0.59
	Female only	70	-0.07 to 0.30
	Male only	66	0.18 to 0.55
	Helper	8	0.04 to 0.86

N = number of effect sizes.

Supplementary Table 6. Random effects and heterogeneity in the analyses

a. Long-term condition and signals

	Random effect	Posterior mean	95% CI	I^2
Begging: Null model	Phylogeny	0.0145	0.0001 to 0.0590	1.2%
	Species	0.0151	0.0002 to 0.0566	1.3%
	Study	0.1245	0.0621 to 0.1977	10.4%
	Units	0.0424	0.0141 to 0.0725	3.5%
	Total			*16.4%*
Begging: Full model	Phylogeny	0.0136	0.0002 to 0.0543	1.1%
	Species	0.0132	0.0002 to 0.0504	1.1%
	Study	0.1220	0.0543 to 0.1902	10.3%
	Units	0.0411	0.0131 to 0.0718	3.5%
	Total			*16.0%*
Structural signals: Null model	Phylogeny	0.0226	0.0002 to 0.0753	2.1%
	Species	0.0229	0.0002 to 0.0647	2.1%
	Study	0.0149	0.0002 to 0.0384	1.4%
	Units	0.0169	0.0047 to 0.0307	1.6%
	Total			*7.2%*
Structural signals: Full model	Phylogeny	0.0328	0.0002 to 0.1060	3.0%
	Species	0.0325	0.0002 to 0.0910	3.0%
	Study	0.0163	0.0003 to 0.0399	1.5%
	Units	0.0087	0.0007 to 0.0183	0.8%
	Total			*8.3%*

b. Feeding and chick information

	Random effect	Posterior mean	95% CI	I^2
Begging: Null model	Phylogeny	0.0108	0.0001 to 0.0400	0.8%
	Species	0.0143	0.0002 to 0.0472	1.1%
	Study	0.0929	0.0326 to 0.1621	7.1%
	Units	0.1840	0.1329 to 0.2371	14.1%
	Total			*23.2%*
Begging: Full model	Phylogeny	0.0104	0.0002 to 0.0380	0.8%
	Species	0.0138	0.0002 to 0.0472	1.1%
	Study	0.0762	0.0149 to 0.1404	5.9%
	Units	0.1888	0.1379 to 0.243	14.6%
	Total			*22.4%*
Structural signals: Null model	Phylogeny	0.0283	0.0002 to 0.1163	2.4%
	Species	0.0211	0.0002 to 0.0863	1.8%
	Study	0.1288	0.0030 to 0.2735	10.7%
	Units	0.0248	0.0003 to 0.0687	2.1%
	Total			*16.9%*
Structural signals: Full model	Phylogeny	0.0282	0.0002 to 0.1190	2.2%
	Species	0.0222	0.0001 to 0.0920	1.8%
	Study	0.1897	0.0132 to 0.2105	15.1%
	Units	0.0157	0.0002 to 0.0477	1.3%
	Total			*20.4%*
Body size: Null model	Phylogeny	0.0163	0.0003 to 0.0402	1.4%
	Species	0.0072	0.0002 to 0.0223	0.6%
	Study	0.0690	0.0421 to 0.0996	5.8%
	Units	0.0893	0.0726 to 0.1081	7.6%
	Total			*15.4%*
Body size: Full model	Phylogeny	0.0085	0.0002 to 0.0233	0.7%
	Species	0.0044	0.0002 to 0.0130	0.4%
	Study	0.0620	0.0380 to 0.0869	5.3%
	Units	0.0875	0.0694 to 0.1055	7.5%
	Total			*14.0%*

Full models correspond to Tables 1 and 2. Models were weighted by study sample size (the number of broods used to calculate the original test statistic). Sample error variance was constrained to 1.

Supplementary Table 7. Heritability of communication strategies

Signal and response strategies	Heritability
Long-term condition and begging	7.16%
Long-term condition and structural signals	36.32%
Feeding and begging	3.60%
Feeding and body size	5.23%
Feeding and structural signals	11.06%

Table reports the percentage of variance in signalling or response strategy that is due to shared phylogeny, from 200 MCMCglmm linear mixed models, including environmental predictability and quality as fixed effects, controlling for repeated measures on studies and species, and weighted by study sample size (the number of broods used to calculate the original test statistic).

Supplementary Table 8. Results of ASReml analyses on environmental and life history influences on parent-offspring communication

Correlation between…	Fixed effects	Mean Wald Statistic	Mean Pr (Chisq)
condition and begging	Reduction strategy	7.40	0.007**
	Environmental quality	7.87	0.02*
	Reduction * Environment	0.52	0.5
condition and structural signal	Reduction strategy	1.02	0.3
	Environmental quality	15.28	0.0005***
	Reduction * Environment	7.48	0.02*
begging and feeding	Reduction strategy	0.05	0.8
	Environmental quality	6.89	0.03*
	Reduction * Environment	1.02	0.6
structural signals and feeding	Reduction strategy	0.72	0.4
	Environmental quality	4.64	0.1
	Reduction * Environment	5.53	0.06.
body size and feeding	Reduction strategy	40.71	2e-10***
	Environmental quality	15.81	0.0004***
	Reduction * Environment	0.32	0.9

Supplementary Table 9. Studies excluded from the meta-analysis

Citation	*Species*	Reason for exclusion
Burford, J. E., Friedrich, T. J. & Yasukawa, K. 1998. Response to playback of nestling begging in the red-winged blackbird, Agelaius phoeniceus. Animal Behaviour, 56, 555-561.	*Agelaius phoeniceus*	Response to begging was at level of whole brood investment, not within-brood food distribution
Davies, N. B., Kilner, R. M. & Noble, D. G. 1998. Nestling cuckoos, Cuculus canorus, exploit hosts with begging calls that mimic a brood. Proceedings of the Royal Society B: Biological Sciences, 265, 673-678.	*Acrocephalus scirpaceus*	Response to begging was at level of whole brood investment, not within-brood food distribution
Kilner, R. M., Noble, D. & Davies, N. 1999. Signals of need in parent-offspring communication and their exploitation by the common cuckoo. Nature, 397, 667-672.	*Acrocephalus scirpaceus*	Response to begging was at level of whole brood investment, not within-brood food distribution
Madden, J. R. & Davies, N. B. 2006. A host-race difference in begging calls of nestling cuckoos Cuculus canorus develops through experience and increases host provisioning. Proceedings of the Royal Society B: Biological Sciences, 273, 2343–51.	*Acrocephalus scirpaceus*	Response to begging was at level of whole brood investment, not within-brood food distribution
Nuechterlein, G. L. 1985. Experiments on the functions of the bare crown patch of downy western grebe chicks. Canadian Journal of Zoology, 63, 464–467.	*Aechmophorus occidentalis*	Condition measure was hunger, not long-term condition
Meade, J., Nam, K.-B., Lee, J.-W. & Hatchwell, B. J. 2011. An experimental test of the information model for negotiation of biparental care. PloS One, 6, e19684.	*Aegithalos caudatus*	Response to begging was at level of whole brood investment, not within-brood food distribution
Li, J., Zhang, Z., Lv, L., Gao, C. & Wang, Y. 2014. Do Parents and Helpers Discriminate between Related and Unrelated Nestlings in the Cooperative Breeding Silver-Throated Tit? Ethology, 120, 159-168.	*Aegithalos glaucogularis*	Response to begging was at level of whole brood investment, not within-brood food distribution
Eldegard, K. & Sonerud, G. a. 2010. Experimental increase in food supply influences the outcome of within-family conflicts in Tengmalm's owl. Behavioral Ecology and Sociobiology, 64, 815-826.	*Aegolius funereus*	Response to begging was at level of whole brood investment, not within-brood food distribution
Santangeli, A., Hakkarainen, H., Laaksonen, T. & Korpim-ki, E. 2012. Home range size is determined by habitat composition but feeding rate by food availability in male Tengmalm's owls. Animal Behaviour, 83, 1115-1123.	*Aegolius funereus*	Response to begging was at level of whole brood investment, not within-brood food distribution

Glassey, B. & Forbes, S. 2003. Why brown-headed cowbirds do not influence red-winged blackbird parent behaviour. Animal Behaviour, 65, 1235–1246.	*Agelaius phoeniceus*	No direct test of parental feeding in response to begging, size, or structural signals
Whittingham, L. & Robertson, R. 1993. Nestling Hunger and Parental Care in Red-Winged Blackbirds. The Auk, 110, 240-246.	*Agelaius phoeniceus*	Condition measure was hunger, not long-term condition
Mock, D. W., Lamey, T. C. & Ploger, B. J. 1987. Proximate and Ultimate Roles of Food Amount in Regulating Egret Sibling Aggression. Ecology, 68, 1760-1772.	*Ardea herodia; Casmerodius albus*	No direct test of the effect of long-term condition on begging or structural signals. Measure was scissoring position, which may conflate begging with size
Granadeiro, J. P., Bolton, M., Silva, M. C., Nunes, M. & Furness, R. W. 2000. Responses of breeding Cory's shearwater Calonectris diomedea to experimental manipulation of chick condition. Behavioral Ecology, 11, 274-281.	*Calonectris diomedea*	Species obligately lay only 1 egg per brood
Quillfeldt, P. & Masello, J. F. 2004. Context-dependent honest begging in Cory's shearwaters (Calonectris diomedea): influence of food availability. Acta Ethologica, 7, 73-80.	*Calonectris diomedea*	Species obligately lay only 1 egg per brood
Troger, I., Masello, J., Mundry, R. & Quillfeldt, P. 2006. Do Acoustic Parameters of Begging Calls of Cory's Shearwaters Calonectris diomedea Reflect Chick Body Condition? Waterbirds, 29, 315-320.	*Calonectris diomedea*	Species obligately lay only 1 egg per brood
Lichtenstein, G. & Dearborn, D. 2004. Begging and short-term need in cowbird nestlings: how different are brood parasites? Behavioral Ecology and Sociobiology, 56, 352-359.	*Dendroica petechia, Turdus rufiventris*	No direct test of parental feeding in response to begging, size, or structural signals
Weimerskirch, H., Prince, P. & Zimmermann, L. 2000. Chick provisioning by the Yellownosed Albatross Diomedea chlororhynchos: Response of foraging effort to experimentally increased costs and demands. Ibis, 142, 103-110.	*Diomedea chlororhynchos*	Species obligately lay only 1 egg per brood
Waugh, S., Weimerskirch, H., Cherel, Y. & Prince, P. 2000. Contrasting strategies of provisioning and chick growth in two sympatrically breeding albatrosees at Campbell Island, New Zealand. The Condor, 102, 804-813.	*Diomedea melanophris*	Species obligately lay only 1 egg per brood
Weimerskirch, H., Mougey, T. & Hindermeyer, X. 1997. Foraging and provisioning strategies of black-browed albatrosses in relation to the requirements of the chick: natural variation and experimental study. Behavioral Ecology,	*Diomedea melanophris*	Species obligately lay only 1 egg per brood

8, 635-643.		
Liu, C.-J., Du, B., Liu, N.-F., Bao, S.-J. & Zhang, S. 2014. Sex-Specific Parental Care Strategies Via Nestling Age: Females Pay More Attention to Nestling Demands than Males Do in the Horned Lark, Eremophila alpestris. Zoological Science, 31, 348–352.	*Eremophila alpestris*	No direct test of parental feeding in response to begging, size, or structural signals
Patterson, C. B., Erckmann, W. J. & Orians, G. H. 1980. An Experimental Study of Parental Investment and Polygyny in Male Blackbirds. American Naturalist, 116, 757–769.	*Euphagus cyanocephalus*	Response to begging was at level of whole brood investment, not within-brood food distribution
Masman, D., Dijkstra, C., Daan, S. & Bult, A. 1989. Energetic limitation of avian parental effort: Field experiments in the kestrel (Falco tinnunculus). Journal of Evolutionary Biology, 2, 435-455.	*Falco tinnunculus*	Response to begging was at level of whole brood investment, not within-brood food distribution
Mand, R., Rasmann, E. & Magi, M. 2013. When a male changes his ways: sex differences in feeding behavior in the pied flycatcher. Behavioral Ecology, 24, 853-858.	*Ficedula hypoleuca*	Response to begging was at level of whole brood investment, not within-brood food distribution
Ottosson, U., Backman, J. & Smith, H. G. 1997. Begging affects parental effort in the pied flycatcher, Ficedula hypoleuca. Behavioral Ecology and Sociobiology, 41, 381-384.	*Ficedula hypoleuca*	Response to begging was at level of whole brood investment, not within-brood food distribution
Sisask, E., M-nd, R., M-gi, M. & Tilgar, V. 2010. Parental provisioning behaviour in Pied Flycatchers Ficedula hypoleuca is well adjusted to local conditions in a mosaic of deciduous and coniferous habitat. Bird Study, 57, 447-457.	*Ficedula hypoleuca*	Response to begging was at level of whole brood investment, not within-brood food distribution
Harris, M. P. 1983. Parent-young communication in the Puffin Fratercula arctica. Ibis, 125, 109-114.	*Fratercula arctica*	Species obligately lay only 1 egg per brood
Rector, M. E., Walsh, C. J., Kouwenberg, A.-L., Fitzsimmons, M. G. & Storey, A. E. 2014. Signals of need and quality: Atlantic puffin chicks can beg and boast. Behavioral Ecology, 25, 496-503.	*Fratercula arctica*	Species obligately lay only 1 egg per brood
Anderson, M. G., Brunton, D. H. & Hauber, M. E. 2010. Reliable Information Content and Ontogenetic Shift in Begging Calls of Grey Warbler Nestlings. Ethology, 116, 357–365.	*Gerygone igata*	Condition measure was hunger, not long-term condition
Romano, A., Caprioli, M., Boncoraglio, G., Saino, N. & Rubolini, D. 2012. With a little help from my kin: barn swallow nestlings modulate solicitation of parental care according to nestmates' need. Journal of Evolutionary Biology, 25, 1703-1710.	*Hirundo rustica*	Response to begging was at level of whole brood investment, not within-brood food distribution

Miller, D. E. & Conover, M. R. 1979. Differential effects of chick vocalizations and billpecking on parental behavior in the ring-billed gull. Auk, 96, 284-295.	*Larus delawarensis*	Response to begging was at level of whole brood investment, not within-brood food distribution
Mathevon, N. & Charrier, I. 2004. Parent-offspring conflict and the coordination of siblings in gulls. Proceedings of the Royal Society B: Biological Sciences (Suppl.), 271, S145-147.	*Larus ridibundus*	Response to begging was at level of whole brood investment, not within-brood food distribution
MacGregor, N. A. & Cockburn, A. 2002. Sex differences in parental response to begging nestlings in superb fairy-wrens. Animal Behaviour, 63, 923-932.	*Malurus cyaneus*	Response to begging was at level of whole brood investment, not within-brood food distribution
McDonald, P. G., Kazem, A. J. N. & Wright, J. 2009. Cooperative provisioning dynamics: fathers and unrelated helpers show similar responses to manipulations of begging. Animal Behaviour, 77, 369-376.	*Manorina melanophrys*	Response to begging was at level of whole brood investment, not within-brood food distribution
Wright, J., McDonald, P. G., te Marvelde, L., Kazem, A. J. N. & Bishop, C. M. 2010. Helping effort increases with relatedness in bell miners, but unrelated helpers of both sexes still provide substantial care. Proceedings of the Royal Society B: Biological Sciences, 277, 437-45.	*Manorina melanophrys*	Response to begging was at level of whole brood investment, not within-brood food distribution
Koenig, W. D. & Walters, E. L. 2012. An Experimental Study of Chick Provisioning in the Cooperatively Breeding Acorn Woodpecker. Ethology, 118, 566-574.	*Melanerpes formicivorus*	Response to begging was at level of whole brood investment, not within-brood food distribution
Thorogood, R., Ewen, J. G. & Kilner, R. M. 2011. Sense and sensitivity: responsiveness to offspring signals varies with the parents' potential to breed again. Proceedings of the Royal Society B: Biological Sciences, 278, 2638-45.	*Notiomystis cincta*	Response to structural signal was at level of whole brood investment, not within-brood food distribution
Gladbach, A., B--er, C., Mundry, R. & Quillfeldt, P. 2009. Acoustic parameters of begging calls indicate chick body condition in Wilson's storm-petrels Oceanites oceanicus. Journal of Ethology, 27, 267-274.	*Oceanites oceanicus*	Species obligately lay only 1 egg per brood
Nordt, A. 2007. Nestling begging strategies in Wilson's storm-petrels (Oceanites oceanicus): Insights from a supplementary feeding experiment. Diploma dissertation. Friedrich-Schiller-Universit-t Jena: Germany.	*Oceanites oceanicus*	Species obligately lay only 1 egg per brood
Quillfeldt, P. 2002. Begging in the absence of sibling competition in Wilson's storm-petrels, Oceanites oceanicus. Animal Behaviour, 64, 579-587.	*Oceanites oceanicus*	Species obligately lay only 1 egg per brood

Ricklefs, R. E. 1992. The roles of parent and chick in determining feeding rates in Leach's storm-petrel. Animal Behaviour, 43, 895-906.	*Oceanodroma leucorhoa*	Species obligately lay only 1 egg per brood
Duriez, O., Weimerskirch, H. & Fritz, H. 2000. Regulation of chick provisioning in the thin-billed prion: an interannual comparison and manipulation of parents. Canadian Journal of Zoology, 78, 1275-1283.	*Pachyptila belcheri*	Species obligately lay only 1 egg per brood
Quillfeldt, P., Everaert, N., Buyse, J., Masello, J. F. & Dridi, S. 2009. Relationship between plasma leptin-like protein levels, begging and provisioning in nestling thin-billed prions Pachyptila belcheri. General and Comparative Endocrinology, 161, 171-8.	*Pachyptila belcheri*	Species obligately lay only 1 egg per brood
Quillfeldt, P., J. Strange, I. & F. Masello, J. 2007. Sea surface temperatures and behavioural buffering capacity in thin-billed prions Pachyptila belcheri: breeding success, provisioning and chick begging. Journal of Avian Biology, 38, 298-308.	*Pachyptila belcheri*	Species obligately lay only 1 egg per brood
Quillfeldt, P., Masello, J. F., Strange, I. J. & Buchanan, K. L. 2006. Begging and provisioning of thin-billed prions, Pachyptila belcheri, are related to testosterone and corticosterone. Animal Behaviour, 71, 1359-1369.	*Pachyptila belcheri*	Species obligately lay only 1 egg per brood
Quillfeldt, P., Poisbleau, M., Mundry, R. & Masello, J. F. 2010. Are acoustical parameters of begging call elements of thin-billed prions related to chick condition? Acta Ethologica, 13, 1-9.	*Pachyptila belcheri*	Species obligately lay only 1 egg per brood
Grieco, F. 2001. Short-term regulation of food-provisioning rate and effect on prey size in blue tits, Parus caeruleus. Animal Behaviour, 62, 107-116.	*Parus caeruleus*	No direct test of parental feeding in response to begging, size, or structural signals
Slagsvold, T., Amundsen, T. & Dale, S. 1995. Costs and benefits of hatching asynchrony in blue tits Parus caeruleus. Journal of Animal Ecology, 64, 563-578.	*Parus caeruleus*	No direct test of parental feeding in response to begging, size, or structural signals
Tripet, F. & Richner, H. 1997. Host responses to ectoparasites: food compensation by parent blue tits. Oikos, 1997, 557–561.	*Parus caeruleus*	No direct test of parental feeding in response to begging, size, or structural signals
Hinde, C. A. & Kilner, R. M. 2007. Negotiations within the family over the supply of parental care. Proceedings of the Royal Society B: Biological Sciences, 274, 53-60.	*Parus major*	Response to begging was at level of whole brood investment, not within-brood food distribution

Hinde, C. A. 2005. Negotiation over offspring care? A positive response to partner-provisioning rate in great tits. Behavioral Ecology, 17, 6-12.	*Parus major*	Response to begging was at level of whole brood investment, not within-brood food distribution
Kim, K.-J., Son, S.-H., Hwang, H.-S. & Rhim, S.-J. 2014. Effect of begging call playbacks on growth of great tit, Parus major, nestlings. Forest Science and Technology, 10, 29-32.	*Parus major*	Response to begging was at level of whole brood investment, not within-brood food distribution
Kolliker, M., Brinkhof, M. W. G., Heeb, P., Fitze, P. S. & Richner, H. 2000. The quantitative genetic basis of offspring solicitation and parental response in a passerine bird with biparental care. Proceedings of the Royal Society B: Biological Sciences, 267, 2127-2132.	*Parus major*	Condition measure was hunger, not long-term condition
Wright, J. & Dingemanse, N. J. 1999. Parents and helpers compensate for experimental changes in the provisioning effort of others in the Arabian babbler. Animal Behaviour, 308, 345-350.	*Parus major*	Response to begging was at level of whole brood investment, not within-brood food distribution
Mock, D. W., Schwagmeyer, P. L. & Parker, G. A. 2005. Male house sparrows deliver more food to experimentally subsidized offspring. Animal Behaviour, 70, 225-236.	*Passer domesticus*	Response to begging was at level of whole brood investment, not within-brood food distribution
Dor, R. & Lotem, A. 2010. Parental effort and response to nestling begging in the house sparrow: repeatability, heritability and parent-offspring co-evolution. Journal of Evolutionary Biology, 23, 1605-12.	*Passer domesticus*	Response to begging was at level of whole brood investment, not within-brood food distribution; Condition measure was hunger, not long-term condition
Schroeder, J., Nakagawa, S., Cleasby, I. R. & Burke, T. 2012. Passerine birds breeding under chronic noise experience reduced fitness. PloS ONE, 7, 1-8.	*Passer domesticus*	Response to begging was at level of whole brood investment, not within-brood food distribution
Schreiber, E. A. 1996. Experimental Manipulation of Feeding in Red-tailed Tropicbird Chicks. Colonial Waterbirds, 19, 45-55.	*Phaethon rubricauda*	Species obligately lay only 1 egg per brood
Draganoiu, T., Nagle, L., Musseau, R. & Kreutzer, M. 2005. Parental care and brood division in a songbird, the black redstart. Behaviour, 142, 1495–1514.	*Phoenicurus ochruros*	No direct test of parental feeding in response to begging, size, or structural signals
Taylor, S. & Perrin, M. 2008. Adaptive hatching hypotheses do not explain asynchronous hatching in Brown-headed Parrots Poicephalus cryptoxanthus. Ostrich, 79, 205–209.	*Poicephalus cryptoxanthus*	No direct test of parental feeding in response to begging, size, or structural signals
Hamer, K. C., Quillfeldt, P., Masello, J. F. & Fletcher, K. L. 2005. Sex differences in provisioning rules: responses of Manx shearwaters to supplementary chick feeding. Behavioral Ecology, 17, 132-	*Puffinus puffinus*	Species obligately lay only 1 egg per brood

137.

Quillfeldt, P., Masello, J. F. & Hamer, K. C. 2004. Sex differences in provisioning rules and honest signalling of need in Manx shearwaters, Puffinus puffinus. Animal Behaviour, 68, 613-620.	*Puffinus puffinus*	Species obligately lay only 1 egg per brood
Riou, S., Chastel, O. & Hamer, K. C. 2012. Parent-offspring conflict during the transition to independence in a pelagic seabird. Behavioral Ecology, 23, 1102–1107.	*Puffinus puffinus*	Species obligately lay only 1 egg per brood
Kilner, R. M., Madden, J. R. & Hauber, M. E. 2004. Brood parasitic cowbird nestlings use host young to procure resources. Science (New York, N.Y.), 305, 877-9.	*Sayornis phoebe*	Response to begging was at level of whole brood investment, not within-brood food distribution
Heist, C. A. 2013. Effects of Variation in Hunger Levels on Begging Behavior of Nestlings and the Provisioning Behavior of Male and Female Eastern Phoebes. Master's Thesis. Eastern Kentucky University: USA.	*Sayornis phoebe*	Response to begging was at level of whole brood investment, not within-brood food distribution; Condition measure was hunger, not long-term condition
Haff, T. M. & Magrath, R. D. 2011. Calling at a cost: elevated nestling calling attracts predators to active nests. Biology Letters, 7, 493-5.	*Sericornis frontalis*	Response to begging was at level of whole brood investment, not within-brood food distribution
Barrios-Miller, N. L. & Siefferman, L. 2013. Evidence that fathers, but not mothers, respond to mate and offspring coloration by favouring high-quality offspring. Animal Behaviour, 85, 1377–1383.	*Sialia sialis*	No direct test of parental feeding in response to begging, size, or structural signals
Jimeno, B., Muriel, J., Perez-Rodriguez, L. & Gil, D. 2014. Sexual Differences in Parental Investment in Response to Parent-Absent Calls. Ethology, 120, 258-265.	*Sturnus unicolor*	Response to begging was at level of whole brood investment, not within-brood food distribution
Wegrzyn, E. & Leniowski, K. 2014. Blackcap Sylvia atricapilla nestlings do not produce begging calls until they are able to escape from predators. Ibis, 1-13.	*Sylvia atricapilla*	No direct test of the effect of long-term condition on begging
Leonard, M. L. & Horn, A. G. 2001. Acoustic signalling of hunger and thermal state by nestling tree swallows. Animal Behaviour, 61, 87–93.	*Tachycineta bicolor*	Condition measure was hunger, not long-term condition
Leonard, M. L., Horn, A. G. & Dorland, A. 2009. Does begging call convergence increase feeding rates to nestling tree swallows Tachycineta bicolor? Journal of Avian Biology, 40, 243-247.	*Tachycineta bicolor*	Response to begging was at level of whole brood investment, not within-brood food distribution

Mainwaring, M. C., Lucy, D. & Hartley, I. R. 2014. Hatching Asynchrony Decreases the Magnitude of Parental Care in Domesticated Zebra Finches: Empirical Support for the Peak Load Reduction Hypothesis. Ethology, 120, 577-585.	*Taeniopygia guttata*	Response to begging was at level of whole brood investment, not within-brood food distribution
Rehling, A., Spiller, I., Krause, E. T., Nager, R. G., Monaghan, P. & Trillmich, F. 2012. Flexibility in the duration of parental care: zebra finch parents respond to offspring needs. Animal Behaviour, 83, 35–39.	*Taeniopygia guttata*	No direct test of parental feeding in response to begging, size, or structural signals
Tanaka, K. D. & Ueda, K. 2005. Horsfield's Hawk-Cuckoo Nestlings Simulate Multiple Gapes for Begging. Science, 308, 2005.	*Tarsiger cyanurus*	Response to begging was at level of whole brood investment, not within-brood food distribution
Phillips, R. A. & Croxall, J. P. 2003. Control of provisioning in grey-headed albatrosses (Thalassarche chrysostoma): Do adults respond to chick condition? Canadian Journal of Zoology, 81, 111-116.	*Thalassarche chrysostoma*	Species obligately lay only 1 egg per brood
Gloag, R. & Kacelnik, A. 2013. Host manipulation via begging call structure in the brood-parasitic shiny cowbird. Animal Behaviour, 86, 101-109.	*Troglodytes aedon, Parus major*	Response to begging was at level of whole brood investment, not within-brood food distribution
Wright, J. 1998. Helpers-at-the-nest have the same provisioning rule as parents-: experimental evidence from play-backs of chick begging. Behavioral Ecology and Sociobiology, 42, 423-429.	*Turdoides squamiceps*	Response to begging was at level of whole brood investment, not within-brood food distribution
Ibanez-Alamo, J. D., Arco, L. & Soler, M. 2011. Experimental evidence for a predation cost of begging using active nests and real chicks. Journal of Ornithology, 153, 801-807.	*Turdus merula*	Response to begging was at level of whole brood investment, not within-brood food distribution
Roulin, A. & Bersier, L.-F. 2007. Nestling barn owls beg more intensely in the presence of their mother than in the presence of their father. Animal Behaviour, 74, 1099-1106.	*Tyto alba*	Response to begging was at level of whole brood investment, not within-brood food distribution

Supplementary Note 1

Example R code: MCMCglmm

```
prior = list(R=list(V = 1, nu = 0.002), G=list(G1 = list(V=1,nu=0.002), G2 = list(V=1,nu=0.002),
G3=list(V=1,nu=0.002)))

model <- MCMCglmm(Z_condition_beg ~ reduction_y_n*environment -1, #1
        random = ~ animal + common_name + study, #2
        prior = prior, #3
        pedigree = full_tree[[i]], #4
        mev = CB_data_beh$variance, #5
        data = CB_data_beh, family = "gaussian", verbose=F, pr=T, slice=T, #6
        nitt=8000000, burnin=2000000, thin=1000) #7
```

#1 Z-transformed correlation coefficient ~ Brood reduction strategy * Environment – Intercept

#2 Controlling for phylogeny, repeated measures on each species, repeated measures on each study

#3 Uninformative prior probability

#4 The phylogenetic tree

#5 Weighted by variance: $(n-3)^{-1}$, where n = number of broods for that effect size

#6 Data set, distribution of Y variable, and model output arguments

#7 8,000,000 iterations, excluding the first 2,000,000 iterations, and thinning every 1000

Supplementary Methods

ASReml Analyses

To confirm the MCMCglmm results, we analysed the data using ASReml-R[1]. MCMCglmm is a Bayesian analysis that moves through parameter space through repeated iterations until the model converges on the best posterior estimate of confidence intervals, which allows the detection of effects with smaller sample sizes than traditional, maximum likelihood statistical tests, which assume larger sample sizes[2-4]. However, *a priori* assumptions about the prior probability distributions of random effects must be made. Those assumptions could potentially bias results. ASReml makes no such assumptions, but gives less accurate confidence intervals for variance components, which could bias estimates of fixed effects' coefficients[2,3]. Employing both techniques allows us to determine whether our results are robust or statistical artefacts. Linear mixed models were run with 250 random trees with an Erickson backbone and 250 with a Hackett backbone[5]. The 500 models were averaged to determine the significance of fixed effects using the Wald test, a pseudo-analysis of variance (Supplementary Table 8).

The only difference between the results of our ASReml and MCMCglmm analyses is that the effect of the environment on the correlation between structural signals and feeding changes from significant (pMCMC=0.007) to non-significant (p=0.098). This may be due to low sample size for this analysis (n = 4 effect sizes in poor environments and n = 6 in good environments), which influences maximum likelihood statistical analyses (such as ASReml) more than Bayesian analyses (such as MCMCglmm)[4]. Bayesian analyses can accommodate a lower sample size to parameters ratio than maximum likelihood models[4].

Supplementary Methods References:

1. Butler, D., Cullis, B., Gilmour, A. & Gogel, B. *ASReml-R Reference Manual.* VSN International Ltd. Hempstead UK (2009).
2. Hadfield, J. D. MCMC Methods for Multi-Response Generalized Linear Mixed Models: The MCMCglmm R Package. *Journal of Statistical Software* **33,** 1–22 (2010).
3. Hadfield, J. D., Krasnov, B. R., Poulin, R. & Nakagawa, S. A Tale of Two Phylogenies: Comparative Analyses of Ecological Interactions. *Am Nat* **183,** 174–187 (2014).
4. Lee, S.-Y,& Song X.-Y. Evaluation of the Bayesian and maximum likelihood approaches in analyzing structural equation models with small sample sizes. *Multivariate Behavioral Research* **39**, 653–686 (2004).
5. Jetz, W. *et al.* Global distribution and conservation of evolutionary distinctness in birds. *Curr. Biol.* **24,** 919–930 (2014).
